# Numerical Study on Heat Transfer Performance of Turbulence Enhancement Configurations for Galinstan Based Mini-Channel Cooling

**DOI:** 10.3390/mi17010083

**Published:** 2026-01-07

**Authors:** Fajing Li, Junxi Han, Zhifeng Wang, Yi Dai, Peizhu Chen

**Affiliations:** 1 Guangzhou Railway Polytechnic, Guangzhou 511300, China; 2 School of Mechatronic Engineering and Automation, Foshan University, Foshan 528000, China; 3 School of Education, City University of Macau, Macau 999078, China

**Keywords:** microchannel, Galinstan, turbulator, flow field, heat transfer

## Abstract

The escalating heat flux density and temperature in highly integrated microelectronic devices adversely affect their reliability and service life, making efficient thermal management crucial for stable operation. This study utilizes Galinstan liquid metal as the coolant to investigate the flow and heat transfer performance in microchannel heat sinks incorporating various turbulator configurations. It is revealed that for microchannels featuring expanded regions, turbulators that create highly symmetric flow fields are preferable due to improved flow distribution. The long teardrop-shaped turbulator provides the best heat transfer performance among all the investigated heat transfer enhancement structures. And this turbulator yields a 13.8–25.9% higher enhancement effectiveness compared to other configurations, at the expense of a 28–41% increase in pressure loss. However, the sudden cross-sectional expansion in the expanded region causes a significant reduction in fluid velocity. Consequently, microchannels with expanded regions and turbulators exhibit a higher bottom surface temperature than the original, straight microchannels, leading to an overall deterioration in heat transfer performance.

## 1. Introduction

The increasing heat flux in highly integrated microelectronic devices leads to a sharp rise in temperature, which compromises their stability and service life [1]. Therefore, efficient removal of the substantial heat generated by these devices is imperative.

Extensive research has been conducted on the fundamental flow and heat transfer characteristics of single-phase convection in microchannel heat sinks, leading to various proposed enhancement methods. Regarding flow channel optimization, Yoomyeong et al. [2] investigated the wicking performance of micropillar configurations using distilled water at different temperatures. Their results indicated that a higher wicking coefficient is achieved with larger pillar diameters, higher temperatures, and smaller gaps, which is attributed to enhanced capillary pressure, reduced viscous resistance, and improved liquid supply. Soleimanikutanaei et al. [3] incorporated transverse microchannels in a heat sink and examined the heat transfer rate at various Reynolds numbers. Eslami et al. [4] numerically investigated the heat transfer performance of ribbed microchannels with and without filleted corners. Their comparative analysis revealed that filleted corners increase the Nusselt number and the Fanning friction factor. Bayrak et al. [5] numerically analyzed the thermal-hydraulic efficiency of microchannel heat sinks with various geometric modifications. They found that cavity and rib configurations significantly improved the heat transfer efficiency owing to enhanced fluid mixing between the core flow and the near-wall region. Zhu et al. [6] conducted a numerical study on microchannel heat sinks with various geometric configurations and found that the combination of water droplet cavities and rib columns significantly enhances overall performance. Han et al. [7] designed topology-optimized heat sinks, reporting a 57.35% reduction in temperature difference compared to a conventional spider web heat sink at Re = 2056.8. Zhai et al. [8] proposed a microchannel featuring a triangular cavity and rib, achieving a 1.25-fold improvement in its heat transfer performance through optimization of the cavity depth and rib height. Lu [9] incorporated periodic merging chambers with diamond-shaped ribs in a microchannel heat sink. This configuration enhanced the heat transfer efficiency by 3.59%, 13.24%, and 6.34% compared to the use of triangular, rectangular, and cylindrical ribs, respectively.

Concerning the working fluid, the thermal conductivity of water (~0.6 W·m^−1^·K^−1^) is generally insufficient for high-power heat dissipation in semiconductors. To enhance the thermophysical properties of water, scholars have introduced nanoparticles such as Al_2_O_3_ [10], Fe_3_O_4_ [11] and TiO_2_ [12], resulting in an increase in thermal conductivity of over 30%. In contrast, the thermal conductivity of liquid metals can exceed 20 W·m^−1^·K^−1^, significantly surpassing that of conventional nonmetallic fluids. Galinstan, a biocompatible room-temperature liquid metal, is increasingly being applied in high-heat-flux dissipation applications.

Scarpa et al. [13] investigated an active magnetic regenerator utilizing Galinstan as the working fluid. This system achieved a 400% increase in cooling capacity compared to a water-based system at an equivalent coefficient of performance. Li [14] investigated the free-surface flow of Galinstan and found that it forms a uniform liquid film on pickled stainless steel surfaces within a specific velocity range. Zhang et al. [15] experimentally demonstrated that a minichannel cooling system using Galinstan achieved a heat dissipation capacity of 300 W·cm^−2^, with substantially lower pressure losses than a water-based system. Chen et al. [16] designed three types of microchannel heat sinks and identified the composite microchannel as having the lowest flow resistance. Guo et al. [17] introduced microencapsulated phase change material slurry into the liquid metal, which reduced the heat sink’s thermal resistance by 59.02%. Numerical simulations by Muhammad [18] indicated that the heat transfer performance of Galinstan is superior in deep, narrow channels compared to shallow, wide ones. Furthermore, a maximum heat dissipation of 158.3 W·cm^−2^ was reached at a flow rate of 0.21 m·s^−1^ and a temperature difference of 40 K. Xiang [19] employed liquid gallium in a micro-jet impingement system for chip cooling, achieving a 29.8% reduction in thermal resistance and a maximum temperature drop of 12.6 K compared to a water-cooled system.

Galinstan offers higher thermal conductivity and lower flow resistance than traditional coolants, while microchannel heat sinks provide smaller geometric dimensions and a larger heat exchange area than conventional heat sinks. Motivated by these advantages, this study aims to synergistically combine this novel heat transfer fluid with a high-efficiency cooling structure to achieve superior cooling performance. To this end, different turbulator configurations were incorporated into the microchannel, and a conjugate heat transfer model was established to analyze the flow characteristics and thermal performance of a Galinstan-based micro-cooler.

## 2. Research Subject and Boundary Conditions

### 2.1. Research Subject

**Figure 1:**
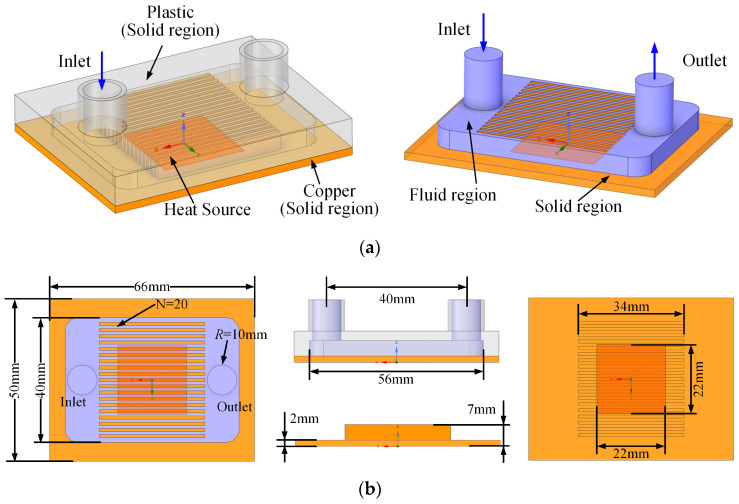
The structure of simulation model: (**a**) Microchannel geometry structure; (**b**) Geometry structure details.

The simulation model consists of three primary components: a thermally conductive substrate integrated with microchannels (solid domain), an upper cover possessing excellent thermal insulation (solid domain), and a fluid domain (indicated by the purple region) for liquid metal flow. The liquid metal enters the microchannel cavity from the fluid inlet, passes through the microchannels, and exits via the fluid outlet. A high-heat-flux chip is positioned in contact with the bottom center of the thermally conductive substrate. Heat from the chip is conducted through the contact area into the substrate, then removed by the liquid metal within the microchannels. In the model, a heat-generating surface equivalent in area to the experimental chip is defined at the substrate bottom. This assumes that the entire heat load from the chip is transferred into the substrate through this interface. To validate the simulation model against the experimental results of Zhang [15], the microchannel geometry from that study is adopted in the present numerical model, as illustrated in Figure 1.

Following model validation, a verified numerical model was established. To balance computational efficiency with resolution of flow and heat transfer details, the geometry was simplified for the main investigation. The liquid metal was assumed to be uniformly distributed at the inlet cavity, resulting in identical flow velocities in each parallel microchannel.

**Figure 2:**
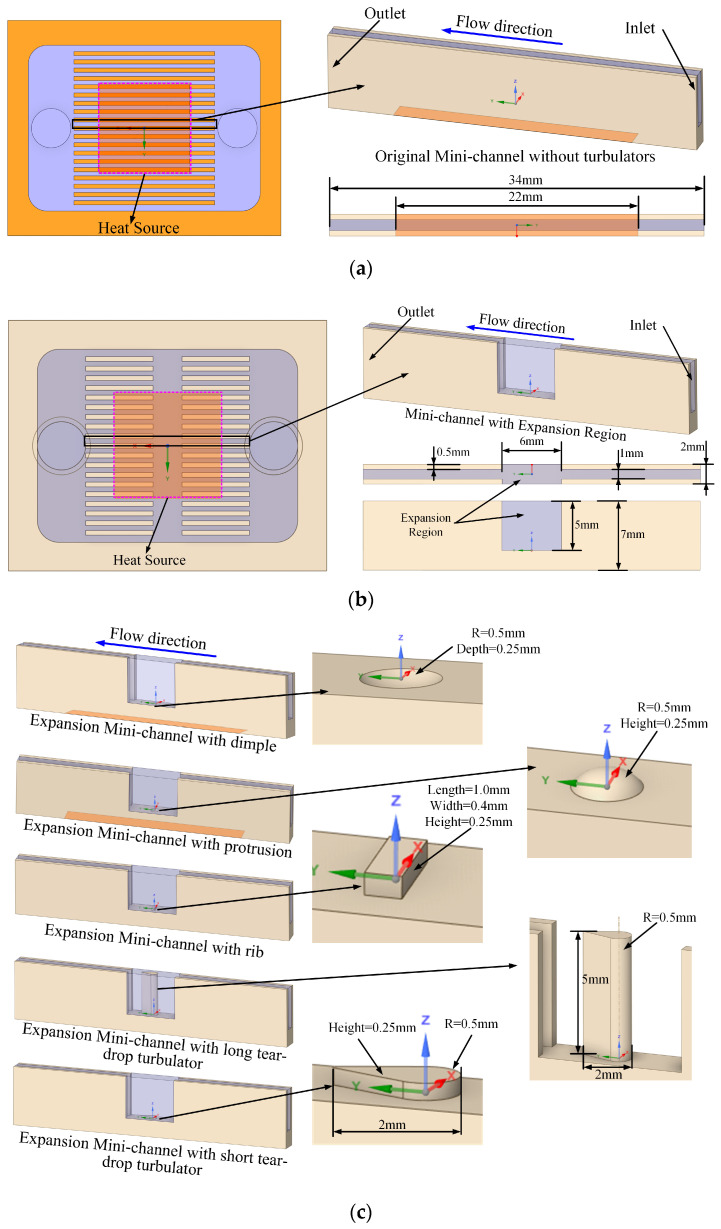
Microchannel parameters with different turbulence configurations: (**a**) Original microchannel; (**b**) Expanded-region microchannel (EM); (**c**) Expanded-region microchannel with turbulence-enhancing configurations.

The baseline configuration without structural modifications is denoted as the original microchannel. Microchannels that feature expanded regions, formed by widening the central sections, are termed expanded-region microchannels (EM). Those incorporating turbulence-enhancing configurations are categorized according to their specific embedded configurations, such as EM with dimple, EM with protrusion, EM with rib, EM with long teardrop turbulator, and EM with short teardrop turbulator. The geometric configurations and dimensional parameters for all microchannel designs studied are presented in Figure 2.

### 2.2. Material Properties and Boundary Conditions

The material properties adopted in the simulation are presented in Table 1.

To enable direct comparison with and validation against the experimental data from Zhang [15], the boundary conditions were aligned with the reported experimental setup. The full-scale simulation model employed the specific inlet temperature, inlet flow rate, wall heat fluxes and inlet-outlet pressure difference. The corresponding parameters are summarized in Table 2.

### 2.3. Data Reduction

To facilitate the comparison of microchannels with different geometric sizes, the inlet boundary conditions are dimensionless.

The Reynolds number is expressed as

(1) $$Re=\frac{m_{s}D}{\left(\mu WH\right)}$$

where *m_s_* is the mass flow rate of inlet, *D* is the hydraulic diameter of channel inlet, *μ* is the kinetic viscosity, *W* and *H* are the width and height of channel inlet.

The local heat transfer coefficient is defined as

(2) $$h=\frac{q}{T_{w}-T_{b}}$$

where *T_w_* is the wall temperature and *T_b_* is the bulk temperature of the working fluid, *q* is the total heat flux of the heated section.

The average Nusselt number is defined as follows

(3) $$Nu=\frac{hD}{\lambda }$$

where *h* is the local heat-transfer coefficient, *D* is the tube hydraulic diameter, and *λ* is the fluid thermal conductivity.

### 2.4. Grid Independence Test and Numerical Model Validation

A three-dimensional, steady-state fluid–structure interaction numerical model was developed and solved using ANSYS (2022 R2) Fluent. Given that the compressibility of the Galinstan liquid metal is negligible compared to that of the gaseous working media, a pressure-based implicit steady solver was employed. For closure of the RANS equations, the widely adopted SST k-ω turbulence model was employed [20]. The SIMPLEC algorithm was used for pressure-velocity coupling, and the PRESTO! scheme was applied for pressure spatial discretization. The computational domain extends from the fluid inlet to the outlet section to eliminate the reversed flow effect.

To evaluate grid independence, a unit channel segment was selected for a mesh sensitivity analysis. Computational grids with varying node counts, generated by adjusting mesh sizes and near-wall refinement parameters, were simulated under identical boundary conditions. This systematic approach assessed the influence of mesh density on the predicted fluid flow and heat transfer characteristics. The corresponding grid numbers are summarized in Table 3.

**Figure 3:**
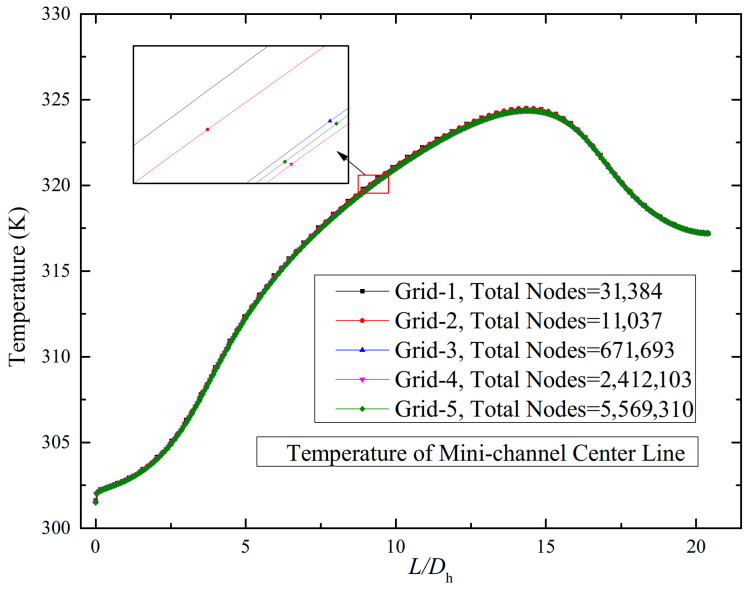
The wall temperature along the centerline of the channel bottom surface under different mesh densities.

The total mass flow rate at the model inlet was set to 0.3296 kg·s^−1^. Assuming a uniform flow distribution across all 20 parallel channels, the mass flow rate per single channel was calculated to be 0.01648 kg·s^−1^. The inlet temperature was set to 301 K, a uniform heat flux of 150.37 W·cm^−2^ was applied at the bottom heating surface of the heat sink. Galinstan was employed as the working coolant. The fluid flow and thermal characteristics obtained under these boundary conditions are presented in Figure 3.

The results indicate that the discrepancies between simulation results obtained with different mesh densities progressively decrease with increasing mesh node count and enhanced near-wall refinement. Specifically, when the number of grid elements increases from 2.41 million to 5.57 million, the resulting discrepancy in the predicted temperature along the channel centerline is merely 0.064%. However, the computational time for 5.57 million grid elements is approximately three times that of 2.41 million grid elements. Therefore, to achieve an optimal balance between numerical accuracy and computational efficiency, the boundary layer refinement methodology and element size parameters of 2.41 million grid elements were selected for all subsequent simulations.

To validate the numerical model, a full-scale microchannel heat sink, identical to the one investigated experimentally by Zhang et al. [15], was adopted as the simulation subject. The geometric configuration and boundary conditions of the model are described in Section 2.1 and Section 2.2, respectively. The simulation results were then compared with the experimentally measured temperature data on the heat sink bottom surface.

**Figure 4:**
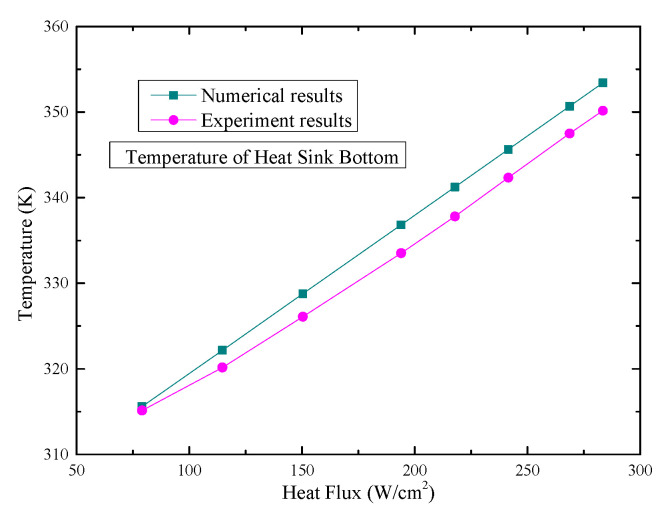
Comparison of numerical results and experimental results.

A quantitative comparison between the simulated and experimentally measured bottom temperatures is presented in Figure 4. As the applied heat flux increases from 79.0 W·cm^−2^ to 283.4 W·cm^−2^, the corresponding relative errors are 0.148%, 0.624%, 0.819%, 0.989%, 1.018%, 0.963%, 0.913%, and 0.929%, respectively. Crucially, all relative errors are below 5%, indicating satisfactory agreement between the numerical model and the experimental results.

## 3. Results and Discussion

### 3.1. Vortex and Flow Field Distributions of Different Turbulator Configurations

The flow field structure is paramount to the efficacy of forced convection heat transfer. Introducing distinct turbulator configurations into a channel generates characteristic flow regimes, which directly modulate the convective heat transfer at the walls. Consequently, the flow fields within microchannels of various configurations are analyzed first.

**Figure 5:**
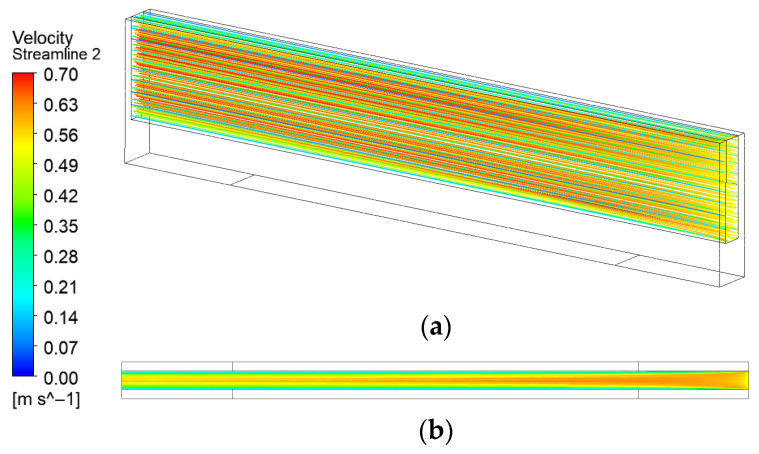
Streamline distribution of original microchannel: (**a**) 3D streamline; (**b**) 2D streamline near the bottom surface.

Figure 5 presents the flow field in the original microchannel without turbulators. Both the three-dimensional and near-bottom two-dimensional streamlines reveal a highly stable, laminar flow with no discernible flow disturbances. According to the principles of forced convection heat transfer, greater turbulence intensity near the heat-exchange surface enhances wall heat transfer efficiency. Consequently, low-turbulence flow inherently limits the convective heat transfer performance of this configuration.

**Figure 6:**
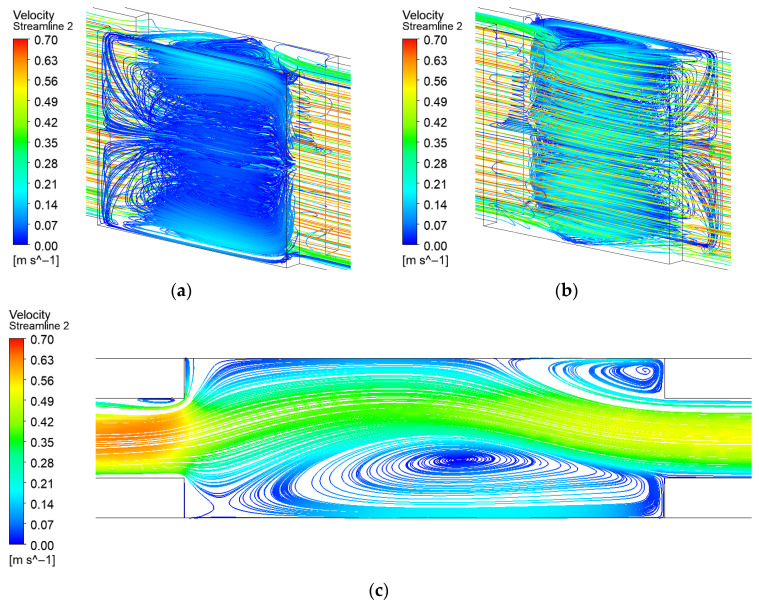
Streamline distribution of expanded-region microchannel (EM): (**a**) Front 3D streamline; (**b**) Back 3D streamline; (**c**) 2D streamline near the bottom surface.

In the expanded-region microchannel without turbulators, highly asymmetric vortices form within the expansion region. As illustrated in Figure 6, the vortex on one side occupies a substantially larger area, leading to a pronounced asymmetry in the flow field. However, these secondary flow vortices are generated by fluid backflow and consequently maintain relatively low velocities, which provides limited enhancement to the heat transfer at the bottom surface.

**Figure 7:**
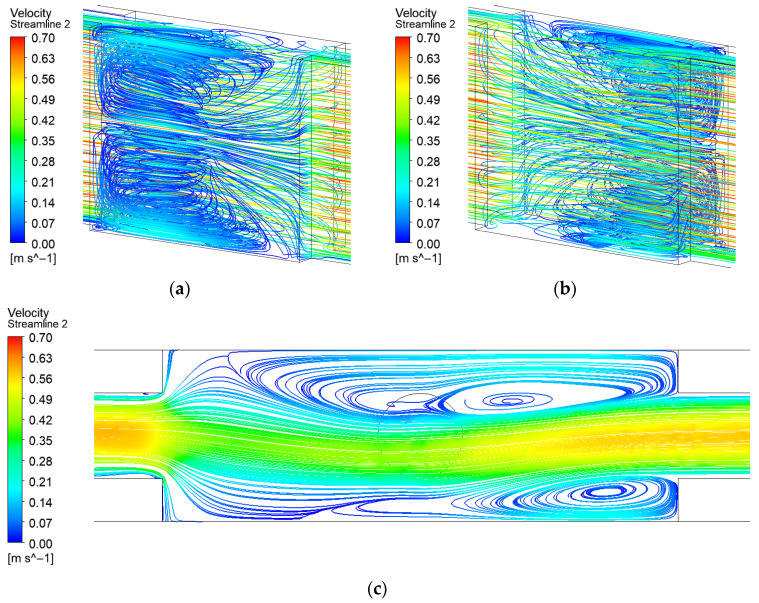
Streamline distribution of EM with dimple: (**a**) Front 3D streamline; (**b**) Back 3D streamline; (**c**) 2D streamline near the bottom surface.

Figure 7 illustrates the streamline distribution within the expanded microchannel (EM) equipped with a dimple turbulator, revealing a markedly asymmetric flow pattern. Moreover, the low-velocity vortex in the near-bottom region occupies a substantially larger area compared to that in the EM without turbulators.

**Figure 8:**
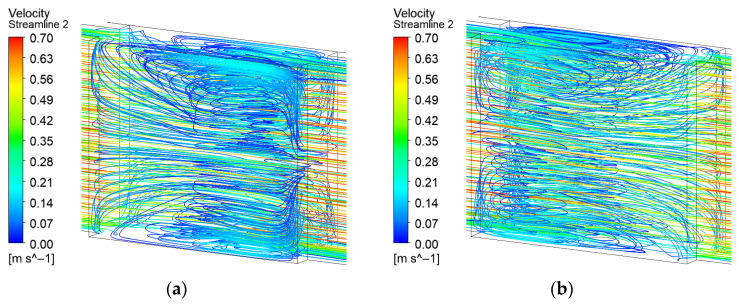

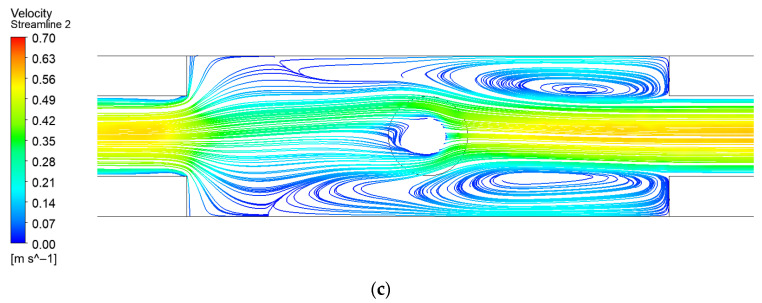
Streamline distribution of EM with spherical protrusion: (**a**) Front 3D streamline; (**b**) Back 3D streamline; (**c**) 2D streamline near the bottom surface.

For the flow channel with spherical protrusion turbulators, the protrusions positioned in the expansion region effectively split the flow along both sides, thereby improving overall flow symmetry, as shown in Figure 8. Compared to both the original microchannel and the expanded-region microchannel with dimple, the spherical protrusion configuration demonstrates significantly enhanced symmetry in the flow field.

**Figure 9:**
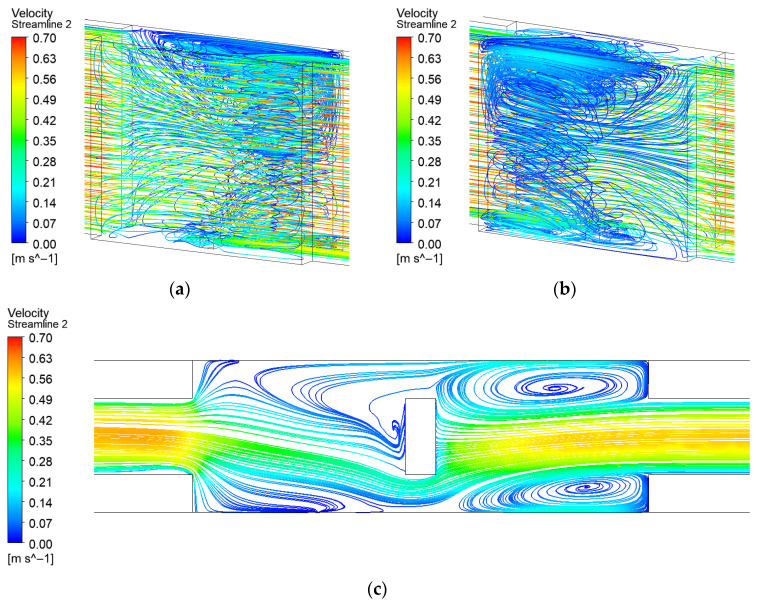
Streamline distribution of EM with rib: (**a**) Front 3D streamline; (**b**) Back 3D streamline; (**c**) 2D streamline near the bottom surface.

Figure 9 illustrates that in the microchannel with rectangular rib turbulators, the flow field near the bottom surface exhibits notable asymmetry. This asymmetry can be attributed to the relatively weak flow separation induced by the rectangular rib geometry. The solid ribs create an obstructive effect, resulting in lower flow velocity in the wake region behind the ribs compared to both the original microchannel and the expanded-region microchannel with dimple.

**Figure 10:**
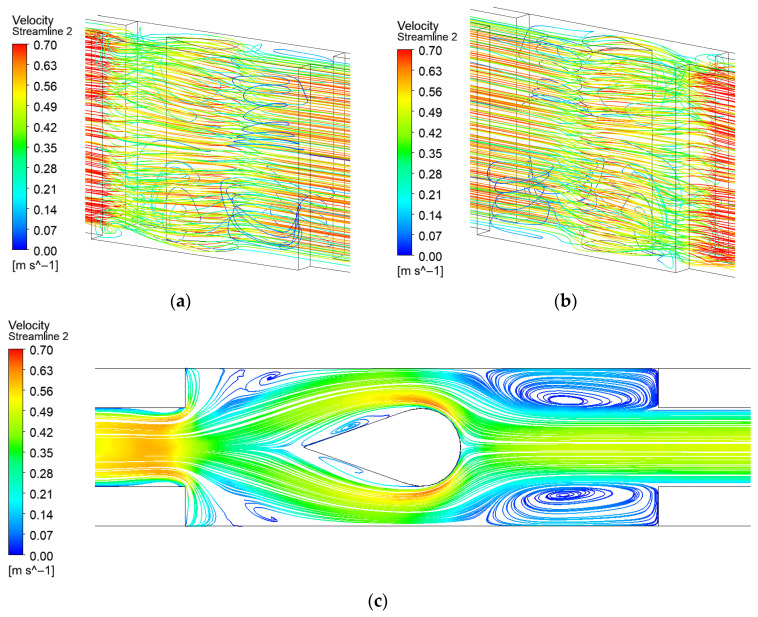
Streamline distribution of EM with long teardrop turbulator: (**a**) Front 3D streamline; (**b**) Back 3D streamline; (**c**) 2D streamline near the bottom surface.

Among all investigated turbulator configurations, the microchannel with a long teardrop turbulator demonstrates the most symmetric flow streamline distribution about the channel centerline, as shown in Figure 10. Two large-scale, low-velocity vortices are formed at the entrance of the expansion segment, while a flow stagnation point emerges at the windward center of the long teardrop turbulator. The flow bifurcates around the turbulator, generating high-velocity regions along both sides, which significantly enhances heat transfer performance. The separated flow streams subsequently impinge on the windward surfaces at the expansion zone outlet, leading to localized flow deceleration. Due to the symmetric boundary conditions, two additional flow stagnation points develop at the 90° right-angle corners of these windward surfaces. Through entrainment with the mainstream, these stagnation points induce secondary flow vortices. The resulting complex flow interaction substantially contributes to the overall improvement in heat transfer efficiency.

**Figure 11:**
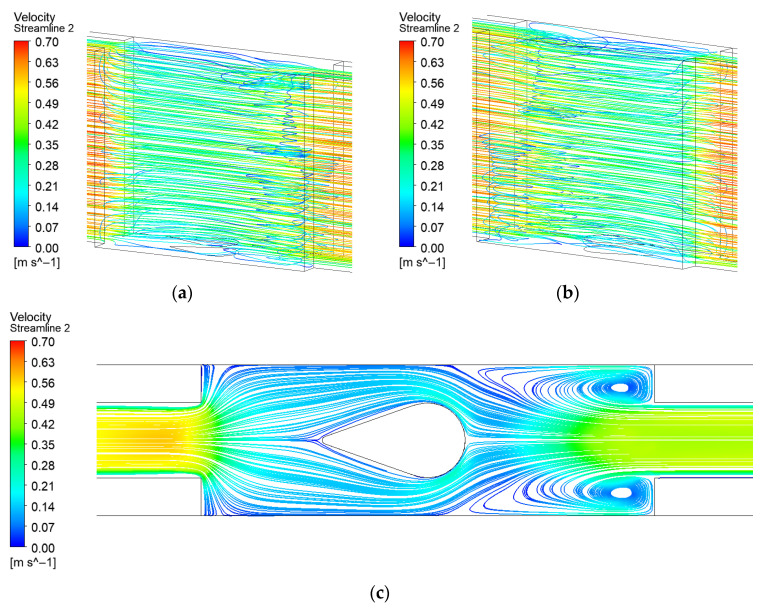
Streamline distribution of EM with short teardrop turbulator: (**a**) Front 3D streamline; (**b**) Back 3D streamline; (**c**) 2D streamline near the bottom surface.

Compared with the long teardrop design, the near-wall flow characteristics in the microchannel with a short teardrop turbulator initially appear similar, as shown in Figure 11. However, its reduced height allows the coolant to predominantly bypass the obstacle from above, resulting in significantly weaker flow acceleration along the turbulator sides. Consequently, the heat transfer enhancement achieved with this design is substantially lower. Furthermore, no pronounced secondary flow vortices form in the 90° corner regions of the windward surface within the expansion zone.

**Figure 12:**
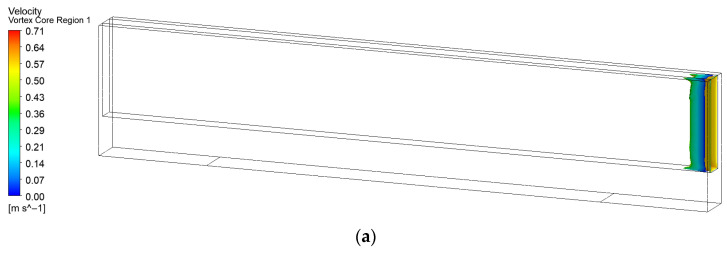

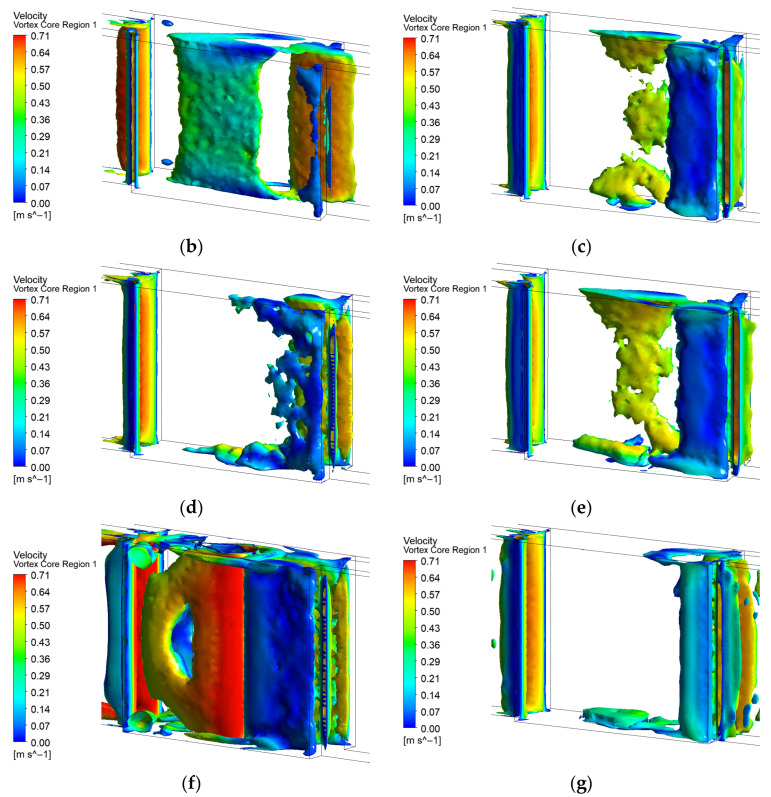
Distribution of vortex cores in microchannels with different configurations: (**a**) Original microchannel; (**b**) Expanded-region microchannel (EM); (**c**) EM with dimple; (**d**) EM with spherical protrusion; (**e**) EM with rib. (**f**) EM with long teardrop turbulator; (**g**) EM with short teardrop turbulator.

Vortex intensity critically influences the behavior of secondary flow vortices. Several methods exist to characterize vortex strength, including streamline analysis, vorticity analysis, the λci method, and the Q-criterion. Based on preliminary experience [21], the λ_2_-criterion was adopted to visualize the vortex cores, enabling intuitive observation of their intensity and influence range. Under an identical λ_2_ iso-value, a vortex core with a larger area and higher local flow velocities indicates a broader influence range and greater strength, which is more conducive to enhancing wall heat transfer efficiency. Furthermore, for forced convection heat transfer, when vortex core dimensions and flow velocities are similar, a closer proximity of the vortex core center to the heat exchange wall contributes more significantly to heat transfer enhancement. The vortex core distributions for microchannels with different turbulator configurations at λ_2_ = −7700 s^−2^ are compared in Figure 12.

As illustrated in Figure 12, only small-scale vortex cores are observed near the inlet of the original smooth microchannel, with no significant vortex structures present elsewhere. Among all the investigated configurations, the long teardrop turbulator generates the most extensive vortex distribution within the microchannel, followed by the expanded-region microchannel without turbulators. The short teardrop turbulator generates the smallest vortex core area. Pronounced flow asymmetry is evident in the expanded-region, dimple, and rectangular rib turbulator channels. In contrast, the flow distributions in channels equipped with the long teardrop, spherical protrusion, and short teardrop turbulators are notably symmetric.

### 3.2. Thermal Performance of the Bottom Wall with Various Turbulator

**Figure 13:**
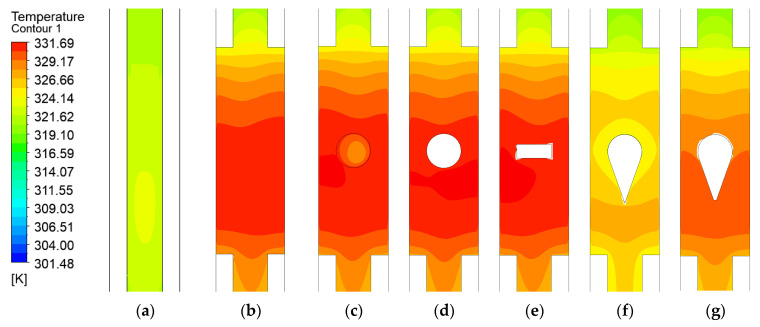
Contour of temperature distribution in microchannels with different configurations: (**a**) Original microchannel; (**b**) Expanded-region microchannel (EM); (**c**) EM with dimple; (**d**) EM with spherical protrusion; (**e**) EM with rib. (**f**) EM with long teardrop turbulator; (**g**) EM with short teardrop turbulator.

As shown in Figure 13, the original microchannel without expansion regions maintains a relatively low bottom surface temperature at locations corresponding to the expansion zones in other configurations. This is primarily because the sudden cross-sectional expansion in other channels reduces the local flow velocity. In contrast, the absence of expansion regions in the original microchannel maintains higher flow velocities and increases the contact area between the fluid and the vertical walls, thereby enhancing overall heat removal and resulting in lower temperatures at the heated surface. This finding provides valuable insights for the thermal enhancement design of channels with expansion regions.

Among all microchannels with expansion regions, the configuration with long teardrop turbulator exhibits the lowest wall temperature, demonstrating the most significant heat transfer enhancement. It is followed in performance by the short teardrop turbulator configuration. Both designs exhibit a well-symmetrical temperature distribution at the bottom surface. In contrast, the maximum temperatures observed in the channels with the dimple, spherical protrusion, and rectangular rib turbulators all exceed that of the baseline expanded-region microchannel without any turbulator.

**Figure 14:**
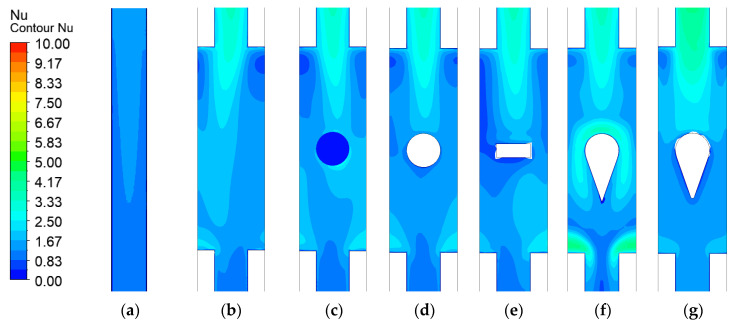
Contour of Nusselt number distribution in microchannels with different configurations: (**a**) Original microchannel; (**b**) Expanded-region microchannel (EM); (**c**) EM with dimple; (**d**) EM with spherical protrusion; (**e**) EM with rib. (**f**) EM with long teardrop turbulator; (**g**) EM with short teardrop turbulator.

Figure 14 presents the Nusselt number (Nu) distribution at the bottom surface. The original microchannel without an expansion region exhibits a relatively low Nu of only 1.265. This is attributed to the lack of an expansion region and consequent absence of flow disturbance, resulting in weaker forced convection effects. In contrast, the introduction of expansion regions, especially when combined with turbulators, significantly enhances the bottom-surface *Nu*, with all such configurations exceeding a value of 1.531. Among them, the long teardrop turbulator achieves the highest *Nu* value of 1.928. This represents a 52.4% improvement over the original microchannel and a 13.8% to 25.9% increase compared to the other turbulator designs, as listed in Table 4.

This improvement primarily results from two mechanisms: Firstly, direct fluid impingement on the windward side of the turbulator generates substantial flow disturbance, enhancing heat transfer; secondly, the two symmetrically separated streams impinge on the contraction zone walls, generating additional strong flow disturbances that further improve thermal performance. For the short teardrop turbulator, however, the heat transfer enhancement is less effective. The reduced turbulator height allows a portion of the fluid to bypass it from above, which diminishes the disturbance intensity in the near-windward region. Similarly, in the contraction zone, incomplete flow separation over the top section limits the overall heat transfer improvement.

**Table 4:** Average *Nu* number at the bottom of microchannels.

Configurations	Average Nusselt Number
Original microchannel	1.265
Expanded-region microchannel	1.691
EM with dimple	1.679
EM with spherical protrusion	1.574
EM with rib	1.531
EM with long teardrop turbulator	1.928
EM with short teardrop turbulator	1.694

### 3.3. Microchannel Pressure Loss with Different Turbulence Configurations

**Figure 15:**
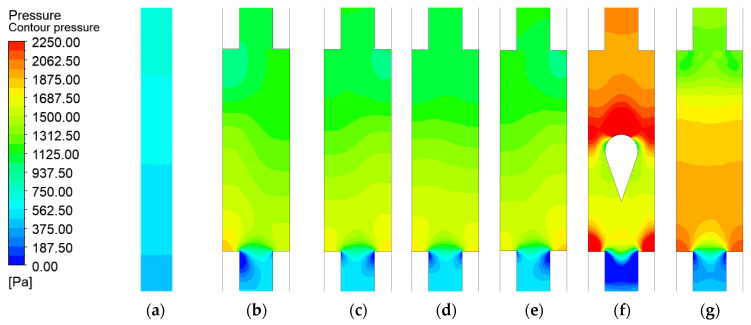
Contour of pressure distribution in microchannels with different configurations: (**a**) Original microchannel; (**b**) Expanded-region microchannel (EM); (**c**) EM with dimple; (**d**) EM with spherical protrusion; (**e**) EM with rib. (**f**) EM with long teardrop turbulator; (**g**) EM with short teardrop turbulator.

Among all investigated configurations, the original design exhibits the lowest pressure loss of 1245.78 Pa and a uniform pressure field distribution, as shown in Figure 15. The incorporation of an expanded region significantly increases the pressure loss to 1588.14 Pa. The highest-pressure loss occurs in the microchannel with the long teardrop turbulator, reaching 2239.94 Pa, which is approximately 28% to 41% higher than that of the other turbulator configurations, as listed in Table 5. As indicated by the preceding flow field analysis, the symmetrical flow structure in the long teardrop turbulator microchannel creates a flow stagnation zone on the windward side of the turbulator, leading to increased pressure. Subsequently, the split flow impinges on the windward side of the contraction section, creating another flow stagnation zone and further increasing the pressure. In contrast, the pressure rise on the windward side is substantially weaker in the short teardrop turbulator microchannel because the liquid can bypass the turbulence structure from above. The microchannels with other turbulence configurations—dimple, spherical protrusion, and rectangular rib exhibit pressure losses between those of the short teardrop turbulator and the expanded-region microchannel, which is consistent with their relatively poorer flow symmetry.

**Table 5:** Pressure loss of microchannels.

Configurations	Pressure Loss [Pa]
Original microchannel	1245.78
Expanded-region microchannel	1588.14
EM with dimple	1595.93
EM with spherical protrusion	1593.55
EM with rib	1608.23
EM with long teardrop turbulator	2239.94
EM with short teardrop turbulator	1748.17

## 4. Conclusions

This study proposed and investigated the thermal performance of a microchannel heat sink using Galinstan liquid metal as the coolant and a thermally conductive substrate as the heat exchange structure. A fluid–structure interaction simulation model was established and validated against experimental results for flow and heat transfer. Subsequently, the microchannel array was simplified to a unit cell with solid domains and a surface heat source, incorporating various turbulence configurations in the channel midsections. The flow and heat transfer processes were analyzed for microchannels with different turbulator configurations. The following conclusions were drawn:

(1) For microchannels with an expansion region, turbulator designs that induce highly symmetric flow fields are preferable. The long teardrop turbulator, which effectively constricts the flow passage to increase coolant velocity and enhance heat transfer, improved thermal performance by 13.8–25.9% compared to other configurations.
(2) The long teardrop turbulator generates a highly symmetric flow field but also induces significant flow stagnation on its windward side and the contraction surface, leading to a pressure loss 28–41% higher than other turbulator configurations. This indicates that the long teardrop-shaped turbulator provides the best heat transfer performance among all the investigated heat transfer enhancement structures. Although, from a numerical perspective, the increase in pressure loss is more pronounced, recent advances in liquid metal pump technology have enabled liquid metal pumps to provide progressively higher pressure. In this study, the authors place more attention on enhancing heat transfer performance.
(3) The sudden expansion of the cross-sectional area substantially reduces fluid velocity. Consequently, all microchannels with expansion regions exhibited a higher bottom surface temperature than the original design without an expansion region, underscoring that the incorporation of expansion regions requires careful consideration in thermal design.

## Figures and Tables

**Table 1:** Thermal and physical properties.

Material	Ga_68_In_20_Sn_12_	Copper	Plastic
Density [kg/m^3^]	6363.2	8978.0	1190.0
Thermal conductivity [W/(m·K)]	23.67 + 0.061 T	387.6	0.15
Specific heat capacity [J/(kg·K)]	366 − 0.70 T	381.0	1464.0
Viscosity [10^−3^ kg/(m·s)]	2.22	/	/

**Table 2:** Simulation boundary conditions.

Numerical Conditions	Model Validation Boundary Conditions	Single Microchannel Boundary Conditions
Inlet temperature [K]	301	301
Inlet mass flow rate [ml/s]	51.8	2.59
Inlet pressure difference [k·Pa]	8.0	8.0
Heat flux density [W/cm^2^]	79.0~283.4	150.37

**Table 3:** Verification of grid independence.

Scheme	Grid Number
Grid-1	31,384
Grid-2	110,372
Grid-3	671,693
Grid-4	2,412,103
Grid-5	5,569,310

## Data Availability

The original contributions presented in the study are included in the article, further inquiries can be directed to the corresponding authors.

## Nomenclature

*D*	hydraulic diameter [m]
*h*	convective heat transfer coefficient [W/(m^2^·K)]
*H*	height of the microchannel [m]
*m_s_*	mass flow rate [kg/s]
*Nu*	averaged Nusselt number
*q*	heat flux [W/m^2^]
*T_w_*	temperature of bottom wall [K]
*T_b_*	temperature of Galinstan [K]
*W*	width of the microchannel [m]
*Re*	Reynolds number
*λ*	thermal conductivity [W/(m·K)]
*µ*	dynamic viscosity [kg/(m·s)]
MC	Expanded-region microchannel
